# Expressed Glycosylphosphatidylinositol-Anchored Horseradish Peroxidase Identifies Co-Clustering Molecules in Individual Lipid Raft Domains

**DOI:** 10.1371/journal.pone.0093054

**Published:** 2014-03-26

**Authors:** Arisa Miyagawa-Yamaguchi, Norihiro Kotani, Koichi Honke

**Affiliations:** 1 Kochi System Glycobiology Center, Kochi University Medical School, Nankoku, Kochi, Japan; 2 Center for Innovate and Translational Medicine, Kochi University Medical School, Nankoku, Kochi, Japan; 3 Department of Biochemistry, Saitama Medical University, Iruma-gun, Saitama, Japan; 4 Department of Biochemistry, Kochi University Medical School, Nankoku, Kochi, Japan; Institut Curie, France

## Abstract

Lipid rafts that are enriched in glycosylphosphatidylinositol (GPI)-anchored proteins serve as a platform for important biological events. To elucidate the molecular mechanisms of these events, identification of co-clustering molecules in individual raft domains is required. Here we describe an approach to this issue using the recently developed method termed enzyme-mediated activation of radical source (EMARS), by which molecules in the vicinity within 300 nm from horseradish peroxidase (HRP) set on the probed molecule are labeled. GPI-anchored HRP fusion proteins (HRP-GPIs), in which the GPI attachment signals derived from human decay accelerating factor and Thy-1 were separately connected to the C-terminus of HRP, were expressed in HeLa S3 cells, and the EMARS reaction was catalyzed by these expressed HRP-GPIs under a living condition. As a result, these different HRP-GPIs had differences in glycosylation and localization and formed distinct clusters. This novel approach distinguished molecular clusters associated with individual GPI-anchored proteins, suggesting that it can identify co-clustering molecules in individual raft domains.

## Introduction

Lipid rafts are membrane microdomains enriched in cholesterol, sphingolipids, glycosylphosphatidylinositol (GPI)-anchored proteins, and Src-family kinases. Their sizes are small ranging mostly between 5 and 20 nm in resting cells, but could be larger on the order of a micron upon stimulation [1], [2]. They are formed by weak interactions between particular membrane lipids and proteins, and display a dynamic property of their association and dissociation [3]. Recent studies have accumulated evidence that lipid rafts serve as a platform in a wide range of important biological events such as signal transduction, cell adhesion, migration, and protein trafficking [4], [5], [6], [7]. In order to elucidate the molecular mechanisms of these events, identification of co-clustering molecules in individual raft domains under a living condition is required. The detergent-resistant floating membrane (DRM) fractionation, which is most commonly employed for isolation of lipid rafts [8], is not suitable for this aim, because the recovered material contains a mixture of heterogeneous microdomains, and therefore it is impossible to determine which molecules in the DRM fraction form an assembly under a living condition. Heterogeneity of membrane microdomains is demonstrated in the previous study using freeze-fracture immunolabeling electron microscopy, in which different types of glycosphingolipids are found to reside in different domains [9]. However, it remains to be elucidated whether distinct molecules are co-clustered with the different types of glycosphingolipids, although the different raft domains contain common raft-associated molecules such as cholesterol, actin filament and Src-family kinases [10].

In mammalian cells, more than 150 membrane proteins are anchored to the membrane via a GPI moiety [11]. GPI is transferred by GPI transamidase to proteins that have a GPI attachment signal sequence at their C-termini in the endoplasmic reticulum (ER) [12]. GPI-anchored proteins are then transported to the plasma membrane through the Golgi apparatus. It has been proposed that GPI functions as sorting signals for selective targeting of GPI-anchored proteins to the secretory and endocytic pathways, which seems to be correlated with their association with the lipid raft domains [8], [13]. The sufficiency of GPI moiety for the preferential localization of GPI-anchored proteins in the lipid rafts has been demonstrated by genetic engineering experiments, in which GPI-anchored green fluorescent protein (GFP) fusion proteins are found to localize in the lipid raft domains [14], [15], [16], [17], [18]. During the intracellular trafficking of GPI-anchored proteins from the ER to the plasma membrane, the structure of GPI moiety is dynamically changed [19]. Recent studies using the mutants of the GPI processing enzymes have revealed that the remodeling of GPI is needed for the intracellular trafficking and association with the lipid rafts of GPI-anchored proteins [19], [20]. Thus, it is widely accepted that the proper GPI structure is a necessary and sufficient condition for the association of GPI-anchored proteins with the lipid rafts.

The GPI attachment signals are poorly conserved on the sequence level, but are composed of four regions: a linker region of about 10 amino acid residues upstream the cleavage site (ω site), a region of small residues (ω to ω+2) including the GPI-attachment site, a short stretch of hydrophilic amino acids, and the C-terminal hydrophobic tail [21]. GPI-anchored GFP fusion proteins having distinct GPI attachment signals are differently sorted depending on their ability of oligomerization [22]. Since only two amino acids (ω and ω–1) are left and the sole amino acid (ω–1) is different in these chimeric proteins after the transfer of GPI, differences in the GPI structures are assumed [22]. It is unknown whether differences in the GPI attachment signals specify the addition of different GPI anchors.

**Figure 1:**
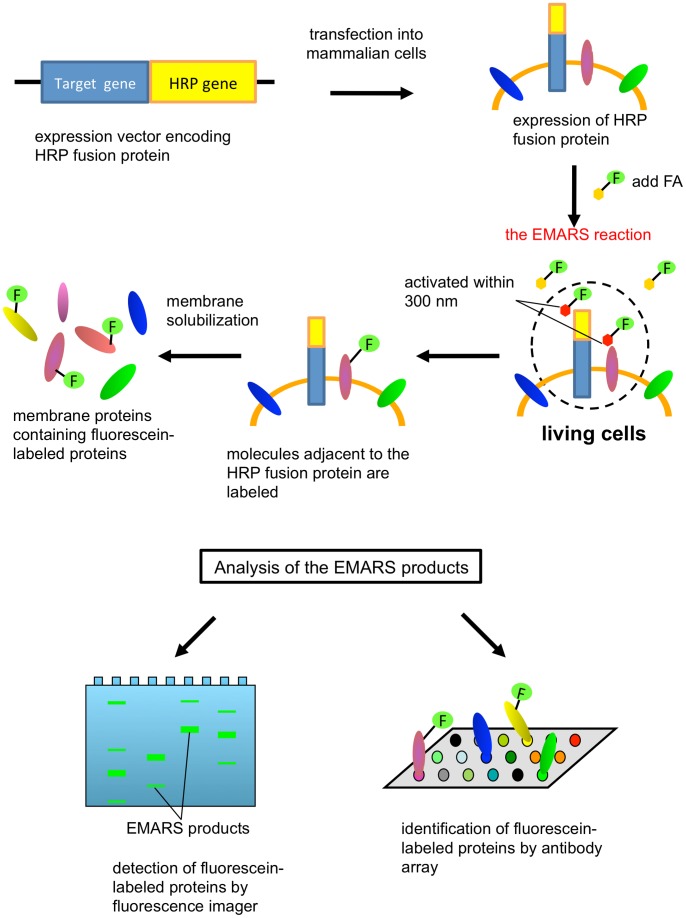
Schematic diagram of the novel EMARS system using HRP expressed by genetic engineering. An expression vector encoding HRP fusion protein is transfected into mammalian cells. Cells expressing an HRP fusion protein are supplemented with fluorescein-conjugated arylazide *(FA)* to initiate the EMARS reaction. After EMARS reaction, membrane proteins are solubilized and the fluorescein-labeled proteins are analyzed using fluorescence imager and antibody array.

Here we describe an approach to identify co-clustering molecules in individual raft domains under a living condition by using the recently developed method termed enzyme-mediated activation of radical source (EMARS), which is featured by radical formation from an arylazide compound by horseradish peroxidase (HRP) [23], [24], [25]. The radicals produced by the EMARS reaction attack and make a covalent bond to the molecules in the vicinity within 300 nm from the HRP set on the probed molecule. The EMARS products can be identified using antibody array [23], [26], [27], [28]. In the present study, the EMARS reaction was performed by the catalysis of intracellularly expressed GPI-anchored HRP fusion proteins (HRP-GPIs) (Figure 1) in substitution for exogenously added HRP-conjugated antibodies that are used previously [23], [26], [27], [28], [29], [30]. By using this approach, we demonstrate that molecular clusters associated with distinct HRP-GPIs, in which the GPI attachment signals derived from human decay accelerating factor (DAF) and Thy-1 were separately connected to the C-terminus of HRP, are different from each other.

## Materials and Methods

### Cell Culture and Antibody Preparation

HeLa S3 cells were cultured in RPMI 1640 medium supplemented with 10% fetal bovine serum (FBS) at 37°C under humidified air containing 5% CO_2_. Goat anti-fluorescein antibody (Rockland) was conjugated to HRP using a peroxidase labeling kit NH2 (Dojindo) following the manufacture’s instruction.

### Lentiviral Vectors and Infections

The DNA fragment encoding the mature region of *Armoracia rusticana* HRP (from Gln^31^ to Ser^338^) was amplified by PCR using the *prxC1a* gene [31] as a template and the primer sets, 5′-CGCGGATCCACAACTTACCCCTACCTTCTACG and 5′-CCGGAATTCCAGAGTTGGAGTTCACCACCC (restriction sites are underlined). The PCR product was digested, purified, and subcloned into *Bam*HI/*Eco*RI-sites of pSecTagA (Invitrogen). Then, the N-terminal signal peptide and the C-terminal GPI attachment signal of GPI-anchored proteins were connected to the corresponding terminus of HRP as follows. Oligonucleotides encoding the GPI attachment signals of human DAF (from Pro^345^ to Thr^381^) and human Thy-1 (from Val^122^ to Leu^161^) were chemically synthesized and separately cloned into *Eco*RV site of pSecTagA-HRP. In addition, DNA fragments encoding the N-terminal signal peptides of DAF (DAFS) and Thy-1 (Thy1S) were cloned into the *Bam*HI site of pSecTagA-HRP. The resulting fusion DNA fragments of DAFS-HRP-DAFGPI and Thy1S-HRP-Thy1GPI were separately subcloned into the *Eco*RV site of pENTR1A no ccdB (Addgene number 17398). The generated pENTR DAFS-HRP-DAFGPI and pENTR Thy1S-HRP-Thy1GPI vectors were recombined in pLenti CMV/TO Puro DEST (Addgene number 17293) using the Gateway LR Clonase enzyme mix (Invitrogen).

Lentiviruses were produced by co-transfection of the different pLenti constructs together with pMD2.G (Addgene number 12259) and psPAX2 (Addgene number 12260) in the HEK293T packaging cell line (RIKEN). After 72 h, supernatants were collected and infections were performed on 5–7.5×10^5^ cells overnight. Appropriate selection was applied 48 hrs later. HeLa S3 cells expressing the tetracycline repressor (TetR) were generated by infection with lentiviruses containing pLenti-CMVtetR Blast (Addgene number 17492). And then, HeLa S3/TetR cells were infected with pLenti CMV/TO Puro lentivirus vectors generated above, yielding the HeLa S3-TetON/HRP-DAFGPI and HeLa S3 TetON/HRP-Thy1GPI cells. For the expression of HRP, the infected cells were incubated with the complete medium supplemented with 1 μg/ml doxycycline for 24 h.

### Vectors and Transfection

The DNA fragment encoding the fluorescent protein *CoralHue* Keima-Red (hmKR) and Asami-Green (hmAG) were amplified from phmKeima-Red-MCLinker (MBL) and phmAG1-MCLinker (MBL) by PCR, respectively. The HRP fragment in pSecTagA/DAFS-HRP-DAFGPI and pSecTagA/Thy1S-HRP-Thy1GPI were replaced with hmKR and hmAG, respectively. The resulting fusion fragments of DAFS-hmKR-DAFGPI and Thy1S-hmAG-Thy1GPI were separately subcloned into the *Eco*RV site of pcDNA3.1/zeo (+) (Invitrogen), yielding pcDNA3.1/DAFS-hmKR-DAFGPI and pcDNA3.1/Thy1S-hmAG-Thy1GPI for analysis of localization of GPI-anchored proteins.

For swapping analysis of the GPI attachment signals, oligonucleotides encoding mutant signal peptides were chemically synthesized. DAF(Thy1_128–132_)GPI was designed as the five amino acids (ω–2 to ω+2 site) of DAF’s GPI attachment signal were replaced with the corresponding five amino acids of Thy-1′s. Thy1(DAF_351–355_)GPI was designed as the five amino acids (ω–2 to ω+2 site) of Thy1’s were replaced with the corresponding five amino acids of DAF’s. These chimeric GPI-anchor attachment signals were separately cloned into *Eco*RV site of pSecTagA-HRP. The Ig κ-chain leader sequence of pSecTagA was used as an N-terminal signal sequence in IgκS-HRP-Thy1_128–132_GPI and IgκS-HRP-DAF_351–355_GPI. These plasmid DNAs were transfected into HeLa S3 cells with Lipofectamine 2000 transfection reagent (Invitrogen) and transiently expressed.

### Western Blotting

Cells were lysed in the SDS-sample buffer, separated by SDS-PAGE and transferred to a PVDF membrane. Immunoblotting was performed with a goat anti-HRP antibody (1∶5000; Jackson ImmunoResearch). HRP-conjugated anti-goat IgG antibody (Santa Cruz Biotechnology) was diluted 10,000-fold and used as a secondary antibody.

### Glycosidase Treatment

Cell lysates were deglycosylated by Peptide-N4-(*N*-acetyl-β-glucosaminyl)asparagine amidase F (*PNGase*) (Sigma-Aldrich), endo-β-N-acetylglucosaminidase H (*EndoH*) (New England Biolabs) or sialidase (Roche Applied Science) treatment. Lysates were incubated with 10% (vol/vol) denaturing buffer (5% SDS, 0.4 M DTT) at 100°C for 10 min. The deglycosylation was performed using 0.05 U/μl PNGaseF, 50 U/μl EndoH or 0.001 U/μl sialidase in the presence of 10% (vol/vol) NP-40 and 50 mM sodium phosphate, pH 7.5 for PNGase, 50 mM sodium citrate, pH 5.5 for EndoH, or pH 4.5 for sialidase, at 37°C overnight.

### Confocal Laser Scan Microscopy

Cells were cultured on 35-mm glass bottom dishes (Iwaki glass) for 24 h with or without 1 μg/ml doxycycline. For the confocal microscopy analysis of the expression of HRP, hmKR or hmAG, each expressed cells were treated with antibodies against HRP (Jackson ImmunoResearch), hmKR (MBL) or hmAG (MBL) at room temperature for 20 min. Then, the cells were treated with Alexa 488-conjugated anti-goat IgG antibody (Invitrogen) for HRP, Alexa 555-conjugated anti-mouse IgG antibody (Invitrogen) for hmKR and Alexa 488-conjugated anti-rabbit IgG antibody (Invitrogen) for hmAG at room temperature for 20 min. After washing with PBS, the cells were fixed with 7.4% formaldehyde-PBS solution at room temperature for 10 min. The cells were gently washed with PBS, and observed with confocal laser scan microscopy (FLUOVIEW FV1000, OLYMPUS).

### Cholesterol Depletion

To suppress synthesis and uptake of cholesterol, cells which had been cultured in RPMI 1640 medium supplemented with 10% FBS were washed with RPMI 1640 alone, and then the medium was replaced with RPMI 1640 medium supplemented with 10% lipoprotein-deficient FCS (Sigma-Aldrich), 10 μM zaragozic acid (Sigma-Aldrich) and 5 μM simvastatin (Sigma-Aldrich). After incubation for 48 h, the cells were washed with Opti-MEM I Reduced-Serum Medium (Invitrogen) and then cultured in Opti-MEM with 10 μM zaragozic acid and 5 μM simvastatin. After incubation for 36 h, the cells were analyzed for lipid rafts fractionation and EMARS reaction as described below.

For removal of cholesterol, cells were treated with 10 mM methyl-beta-cyclodextrin (MβCD) (Sigma-Aldrich) at 37°C for 3 h before analysis.

### Isolation of Lipid Rafts

Cells cultured in 6 cm culture dishes were lysed on ice for 20 min in 400 μl of 1% TritonX-100 in TBS buffer (25 mM Tris-HCl, pH 7.5, 150 mM NaCl) and homogenized (10 strokes) with a loose fitting Dounce homogenizer. The homogenates were mixed with 400 μl of 80% sucrose prepared in TBS buffer and placed on the bottom of a centrifuge tube. The samples were sequentially overlayed with 800 μl of 30% sucrose and 800 μl of 5% sucrose in TBS buffer and centrifuged at 50,000 rpm in TL-100 centrifuge (Beckman Coulter) for 20 h. Fractions (200 μl each) were collected from the top to the bottom and subjected to Western blot analysis.

### Phosphatidylinositol-specific Phospholipase C (PI-PLC) Treatment and Flow Cytometry

Cells were treated with or without 2 IU/ml of phosphatidylinositol-specific phospholipase C (PI-PLC, Molecular Probes) in Opti-MEM I Reduced-Serum Medium (Invitrogen) for 1 h at 37°C. Following the incubation, cells were washed twice in ice-cold PBS, transferred into a plastic tube, and reacted with a goat anti-HRP antibody at 4°C for 30 min. After washing with PBS, cells were treated with Alexa 488-conjugated anti-goat IgG antibody (Invitrogen) at 4°C for 30 min. Cell surface fluorescence was measured by flow cytometry using a FACScan (Becton-Dickinson).

### HRP Activity Assays

Peroxidase activity tests for HRP were performed with a classical peroxidase assay, 2,2′-Azino-bis(3-ethylbenzothiazoline-6-sulfonic acid) (ABTS) and hydrogen peroxide. HRP-DAFGPI or HRP-Thy1GPI-introduced HeLa S3 cells were incubated with *(+Dox)* or without *(–Dox)* doxycycline. Cell lysates (7.5 μg total protein) were mixed with 100 μL of ABTS solution (Sigma-Aldrich) in a 96-well plate and incubated for 10 min at 37°C. Then absorbance at 405 nm was measured with a SpectraMax plate reader (Molecular Devices) at room temperature.

### The EMARS Reaction and Detection of the EMARS Products

Cells were grown in the complete media supplemented with or without 1 μg/ml doxycycline. The EMARS reaction and detection of EMARS products were performed as described previously [30]. Briefly, the cultured cells were incubated with 0.1 mM fluorescein-conjugated arylazide (FA) in PBS at room temperature for 15 min in dark. The cell suspension was then homogenized through a syringe needle to break the plasma membranes and centrifuged at 800 g for 5 min. The supernatant was subsequently centrifuged at 20,000 g for 15 min to precipitate the plasma membrane fractions. After solubilization with the NP-40 lysis buffer (20 mM Tris-HCl (pH 7.4), 150 mM NaCl, 5 mM EDTA, 1% NP-40, 1% glycerol), the samples were subjected to SDS-PAGE (10% gel, under non-reducing conditions), and were subsequently analyzed using LAS-4000 Bio-imaging analyzer (Fuji Film) equipped with blue light and Y515-Di filter under fluorescence mode for FA detection. In order to test the effect of the difference in the *N*-glycan types on cluster formation, cells were treated with 20 μM swainsonine, an inhibitor of Golgi α-mannosidase II for 72 h prior to the EMARS reaction.

### RTKs Array Analysis

A total of 10 μg of the EMARS products were applied to a Proteome Profiler Human Phospho-RTK array (R&D Systems) following the manufactures instrument. After washing, the array was stained with HRP-conjugated anti-fluorescein antibody (0.1 μg/ml) and developed with an Immobilon Western Chemiluminescent HRP Substrate (Millipore). The detailed array coordinates were shown in the manufacture web page (RTK array: http://www.rndsystems.com/pdf/ary001.pdf).

### Velocity Gradients

Cells were grown to confluency in 10 cm dishes, washed in PBS and lysed on ice for 30 min in 20 mM Tris, pH 7.4, 100 mM NaCl, 0.4% SDS and 0.2% TtitonX-100. A sucrose density gradient (5–30%) was layered into a centrifuge tube and the lysate was layered over the 5% part of the gradient. After ultracentrifugation at 45,000 rpm for 16 h, fractions of 1 ml were collected from the top (fraction 1) to the bottom (fraction 10) of the gradients. HRP-GPIs were detected by Western blotting using an anti-HRP antibody.

## Results

### Expression of the GPI-anchored HRPs in the Lipid Rafts of the Plasma Membrane in Human Cells

**Figure 2:**
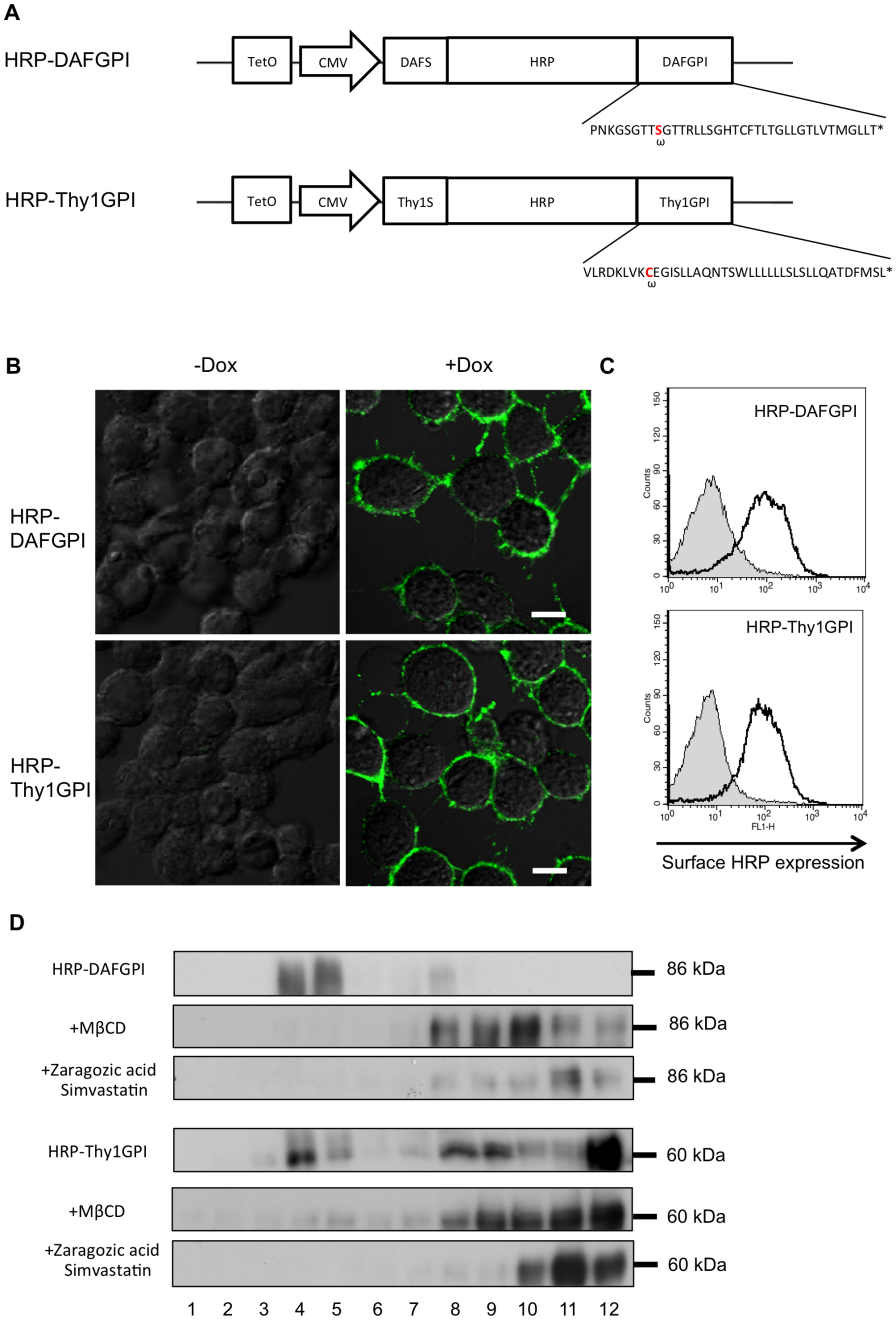
Expression of GPI-anchored HRP in the lipid rafts of the plasma membrane in HeLa S3 cells. *(A)* Schematic representation of the constructs used in this study. HRP-DAFGPI and HRP-Thy1GPI encode HRP fusion proteins connected with the N-terminal signal peptide and the C-terminal GPI attachment signal of human DAF or Thy1, respectively. Their expressions are driven by the doxycycline-dependent TetO promoter combined with CMV promoter. The DAF and Thy1 GPI attachment signal sequences included in the fusion proteins are shown. The *ω-site* is shown in *red*. *Asterisk* indicates the stop codon. *(B)* Cell surface expression of HRP-DAFGPI and HRP-Thy1GPI on HeLa S3 cells was analyzed by confocal laser scan microscopy. Cells were incubated with (+*Dox)* or without (–*Dox*) doxycycline and then reacted with an anti-HRP antibody. Bars = 10 μm. *(C)* PI-PLC susceptibility of HRP-GPIs. HRP-DAFGPI and HRP-Thy1GPI cells were incubated with (*shaded)* or without (*unshaded)* PI-PLC. Cell surface HRP was stained with an anti-HRP antibody and analyzed by flow cytometry. *(D)* The DRM fractionation of HRP-GPIs. HeLa S3 cells expressing HRP-DAFGPI or HRP-Thy1GPI were homogenized in 1% Triton X-100 containing buffer and subjected to sucrose-density ultracentrifugation. Cells were treated with (*+MβCD, +Zaragozic acid and Simvastatin)* or without 10 mM MβCD, 10 μM zaragozic acid and 5 μM simvastatin before homogenize. Aliquots of each fraction were analyzed by Western blotting using anti-HRP antibody. The numbers are ordered from the top to the bottom of the centrifuge tube.

In order to construct two kinds of HRP-GPI, HRP-DAFGPI and HRP-Thy1GPI, the coding region of the *Armoracia rusticana C1a* gene encoding HRP was separately inserted between the DNA fragments encoding the N-terminal signal peptides and the C-terminal GPI-attachment signals of human decay accelerating factor (DAF) (Pro^345^-Thr^381^) and human Thy-1 (Val^131^-Leu^161^), respectively (Figure 2A). Both HRP-GPI constructs were expressed under the Tet-On transcriptional control system [32], [33], because cells that constitutively express HRP-GPI died during selection. The HRP-GPI vectors were separately transfected into HeLaS3 cells and stable transfectants were isolated in the absence of inducer.

When an inducer, doxycycline was added to the culture medium (*+Dox*), HRP was robustly expressed on the cell surface in both HRP-GPI cases in an immunocytochemistry analysis (Figure 2B). To confirm that the expressed HRPs were anchored by GPI, susceptibility to phosphoinositide phospholipase C (PI-PLC) was tested by flow cytometry. PI-PLC cleaves the phosphate-glycerol bond in the GPI moiety and releases GPI-anchored proteins from the membrane [34]. After treatment with PI-PLC, the cell surface expression of HRP disappeared in the HRP-DAFGPI and HRP-Thy1GPI expressing cells (Figure 2C), indicating that both HRP constructs were anchored by GPI. Since GPI-anchored proteins are known to be localized at the lipid rafts, we further investigated the issue of whether the HRP-GPIs are recovered in the detergent-resistant floating membrane (DRM) fraction [8]. As expected, both HRP-GPI proteins were recovered in the DRM fraction (fractions 3–5 in Figure 2D), whereas some HRP-Thy1GPI was recovered in the non-raft fractions. To confirm whether the HRP-GPIs were a lipid raft component, effects of cholesterol depletion on the DRM fractionation were investigated. Cholesterol depletion was accomplished by two ways: one being suppression of synthesis and uptake of cholesterol; the other being removal of cholesterol from membranes. To suppress cholesterol synthesis, cells were treated with zaragozic acid, an inhibitor of squalene synthase and simvastatin, an inhibitor of HMG-CoA reductase. For removal of cholesterol, cells were treated with MβCD. As shown in Figure 2D, both of the HRP-DAFGPI and the HRP-Thy1GPI recovered in the DRM fraction were considerably reduced after the MβCD or the zaragozic acid and simvastatin treatment. These findings clearly demonstrate that both GPI-anchored HRPs were expressed in the lipid rafts of the plasma membrane in human cells.

### Difference in N-glycosylation of the GPI-anchored Proteins with Distinct GPI-attachment Signals

**Figure 3:**
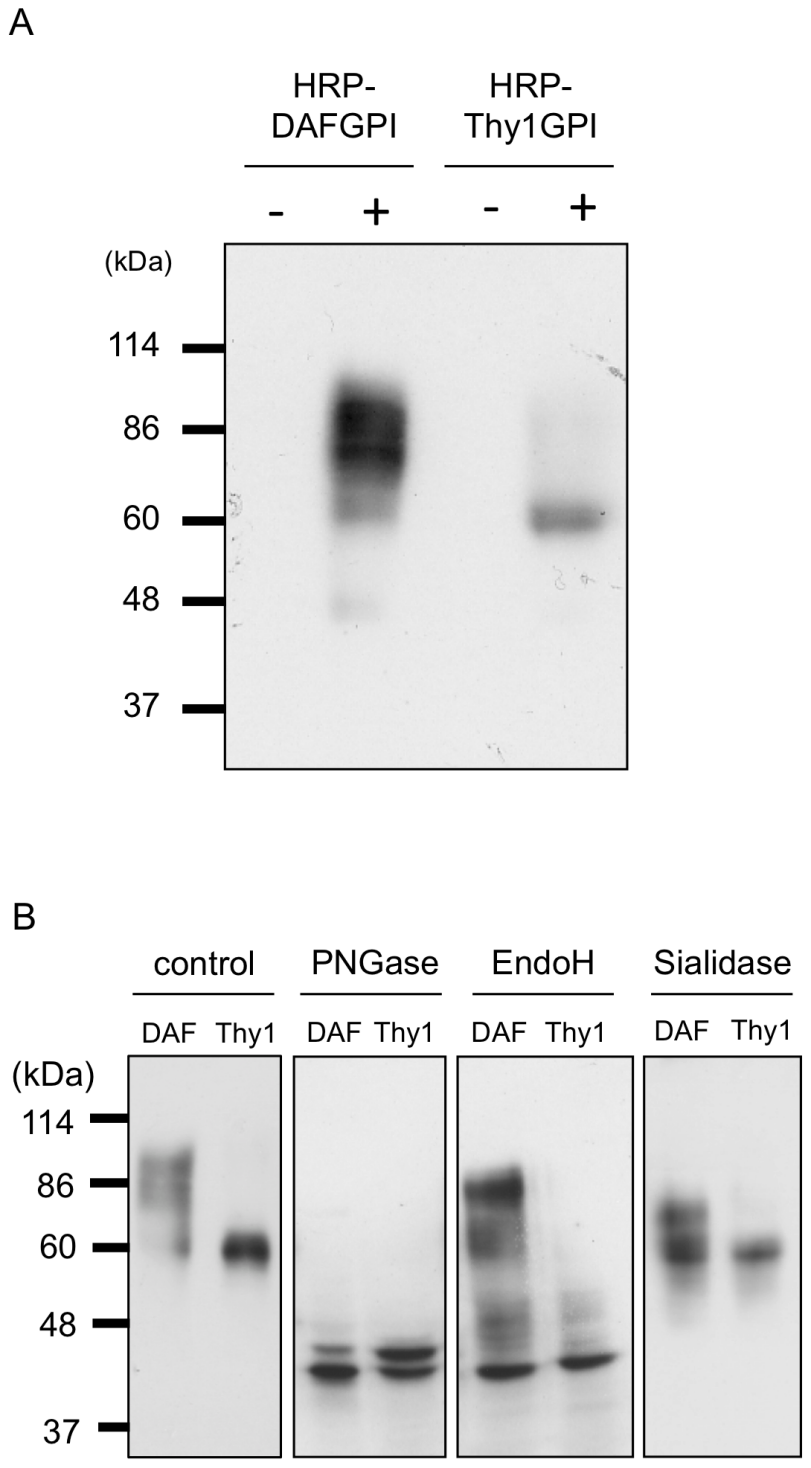
Difference in *N*-glycosylation of HRP-DAFGPI and HRP-Thy1GPI. *(A)* HRP-DAFGPI or HRP-Thy1GPI-introduced cells were incubated with (+) or without (−) doxycycline. Cell lysates were subjected to Western blotting using anti-HRP antibody. *(B)* HRP-DAFGPI *(DAF)* and HRP-Thy1GPI *(Thy1)* proteins expressed in the presence of doxycycline were untreated (*control*) or treated with PNGase, EndoH or sialidase, and analyzed by Western blotting with an anti-HRP antibody.

In order to characterize HRP-DAFGPI and HRP-Thy1GPI molecules biochemically, western blotting was performed using an anti-HRP antibody. As shown in Figure 3A, the molecular size of HRP-DAFGPI was heterogeneous around 86 kDa, while HRP-Thy1GPI was homogeneous at 60 kDa. This result implied the difference in the glycosylation between HRP-DAFGPI and HRP-Thy1GPI, and prompted us to analyze the glycosylation types of these two HRP-GPIs based on sensitivity to some glycan hydrolases.

Peptide-N4-(*N*-acetyl-β-glucosaminyl)asparagine amidase F (*PNGase*) cleaves all types of *N*-glycans including complex type, while endo-β-N-acetylglucosaminidase H (*EndoH*) acts only on high mannose and hybrid type *N*-glycans. Both HRP-GPIs were deglycosylated with PNGase F, resulting in the reduction of molecular mass to the same sizes of 43 and 40 kDa (Figure 3B, *PNGase panel*). However, the 86 kDa molecule of HRP-DAFGPI was resistant to EndoH, but sensitive to sialidase as its apparent size was reduced to approximately 75 kDa (Figure 3B, *DAF lanes in EndoH and Sialidase panels*). In contrast, the 60 kDa molecule of HRP-Thy1GPI was sensitive to EndoH, but resistant to sialidase (Figure 3B, *Thy1 lanes in EndoH and Sialidase panels*). These results indicate that HRP-DAFGPI carries complex type *N*-glycans, while HRP-Thy1GPI possesses high mannose type *N*-glycans. Thus, HRP-DAFGPI and HRP-Thy1GPI undergo different glycosylation in spite of the sameness of the peptide moiety except for the C-terminal oligopeptide region.

### Difference in Localization of GPI-anchored Proteins with Distinct GPI Attachment Signals

**Figure 4:**
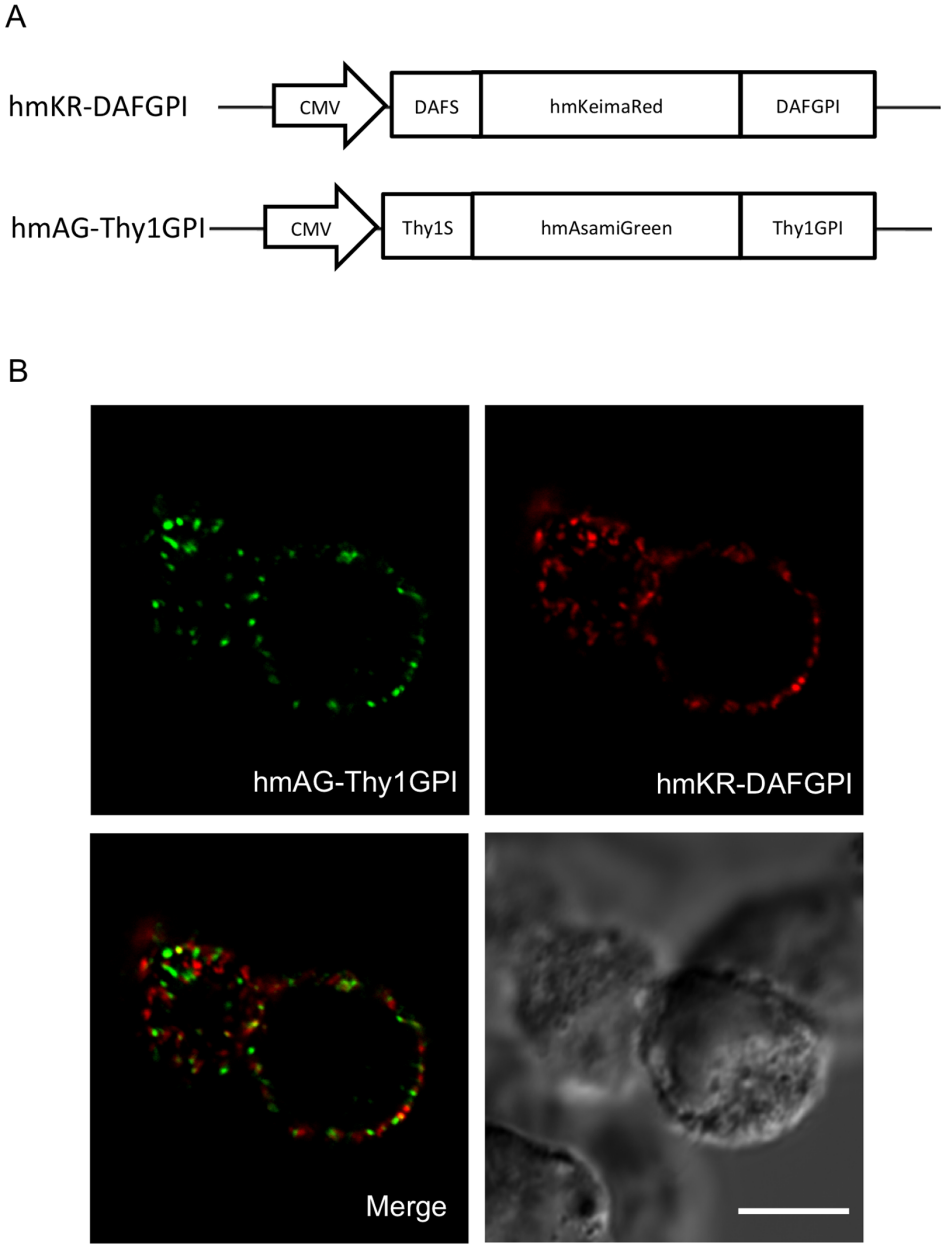
Difference in localization of GPI anchored fluorescent proteins with distinct GPI-attachment signals. *(A)* The constructs of two GPI anchored fluorescent proteins, hmKR-DAFGPI and hmAG-Thy1GPI. In hmKR-DAFGPI, *hmKeimaRed* was fused to the N-terminal signal peptide (*DAFS*) and the C-terminal GPI-attachment signal (*DAFGPI*) of DAF. In hmAG-Thy1GPI, *hmAsamiGreen* was connected with the N-terminal signal peptide (*Thy1S*) and the C-terminal GPI-attachment signal (*Thy1GPI*) of Thy-1. *(B)* The hmKR-DAFGPI and hmAG-Thy1GPI constructs were simultaneously expressed in HeLa S3 cells. Cells were stained with antibodies against hmKR (*red*) and hmAG (*green*) and observed with a confocal microscopy. Bars = 10 μm.

Next we investigated the issue of whether GPI-anchored proteins with different GPI attachment signals are co-localized in the plasma membrane or not. To this end, two exogenous fluorescent proteins fused with distinct GPI attachment signals, hmKeima-Red with the DAF’s GPI attachment signal (hmKR-DAFGPI) and hmAsami-Green with the Thy-1′s GPI attachment signal (hmAG-Thy1GPI) (Figure 4A), were simultaneously expressed in HeLa S3 cells. Confocal laser scan microscopy demonstrated punctate distribution of hmAG-Thy1GPI and hmKR-DAFGPI in the plasma membranes, and their localizations were mutually exclusive (Figure 4B).

### The Expressed HRP-GPIs are Able to Catalyze the EMARS Reaction

**Figure 5:**
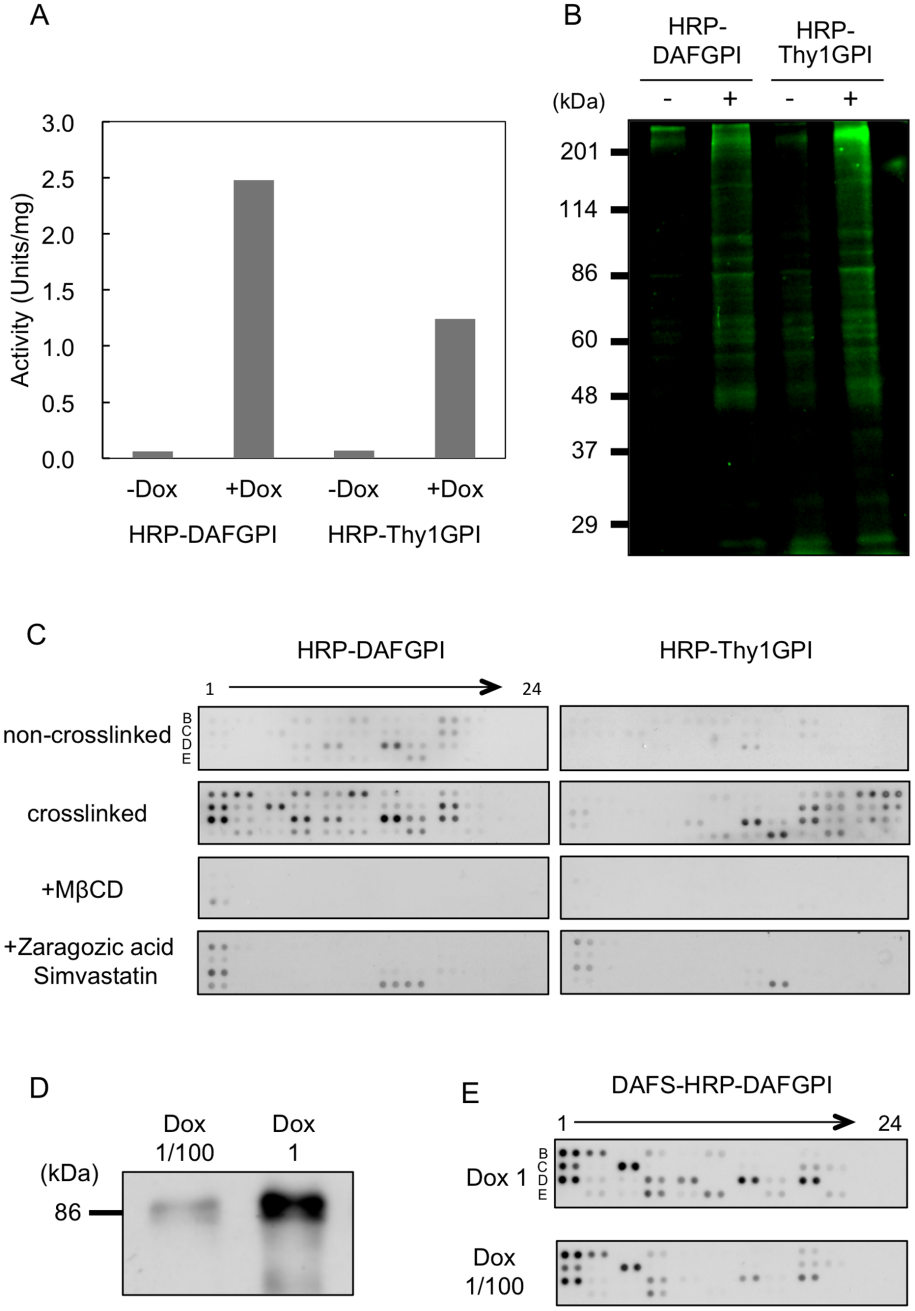
The EMARS reaction catalyzed by the expressed HRP-GPIs and identification of co-clustered molecules with HRP-GPIs. *(A)* Peroxidase activity levels of the expressed HRP-DAFGPI and HRP-Thy1GPI. The values were obtained with the ABTS assays as described in Materials and methods. *(B)* Fluorescence detection of fluorescein-labeled EMARS products. The EMARS reaction was performed using FA as a labeling reagent in HRP-DAFGPI or HRP-Thy1GPI-transfected cells, which had been incubated with *(+)* or without *(–)* doxycycline. After EMARS reaction, 10 μg of microsome proteins were subjected to SDS-PAGE and analyzed by a LAS-4000 fluorescence imager. *(C)* Identification of the fluorescein-labeled EMARS products by antibody array analysis. HeLa S3 cells that express HRP-DAFGPI or HRP-Thy1GPI were treated with *(crosslinked)* or without *(non-crosslinked)* an anti-HRP antibody and subjected to the EMARS reaction. Cells were treated with (*+MβCD, +Zaragozic acid and Simvastatin)* or without 10 mM MβCD, 10 μM zaragozic acid and 5 μM simvastatin before crosslinking. The EMARS products (10 μg total protein) were applied to a RTKs antibody array and fluorescein-labeled proteins were detected with an anti-fluorescein antibody. *(D, E)* Effects of expression level of HRP-GPI on the clustering. (*D*) HRP-DAFGPI -introduced cells were treated with 1 μg (*Dox 1)* or 10 ng (*Dox 1/100)* doxycycline. Cell lysates were subjected to Western blotting using anti-HRP antibody. (*E*) Identification of the fluorescein-labeled EMARS products by the antibody array analysis. HeLa S3 cells that express HRP-DAFGPI were treated with an anti-HRP antibody and subjected to the EMARS reaction. Cell membrane extracts (Dox 1; 10 μg total protein, Dox 1/100; 40 μg total protein) were applied to a RTKs antibody array and the EMARS reaction products were detected with an anti-fluorescein antibody.

In order to investigate the issue of whether the expressed HRP-GPIs are functional or not, peroxidase activity was assayed in the HRP-GPIs expressing cells. As a result, peroxidase activity was significantly increased in the HRP-DAFGPI and HRP-Thy1GPI expressing cells (*+Dox*) as compared to the control cells (*–Dox*) (Figure 5A). This result indicates that the expressed HRP-GPIs include the prosthetic group, heme to function normally.

Then we investigated the issue of whether the expressed HRP-GPIs catalyze the EMARS reaction, in which HRP catalyzes conversion of arylazide group to nitrene radical [23]. The EMARS reaction was performed by incubating with fluorescein-conjugated arylazide in the living cells where HRP-DAFGPI or HRP-Thy1GPI had been stably transfected. When the expression of HRP-GPIs was induced by doxycycline (*+Dox*), a number of fluorescein-labeled bands were detected in both HRP-GPIs expressing cells on a LAS-4000 fluorescence imager while only faint bands that were labeled by unknown endogenous enzyme(s) were seen in the uninduced cells (*–Dox*) (Figure 5B). This result indicates that HRP forcedly expressed in mammalian cells by genetic engineering is able to catalyze the EMARS reaction.

### GPI-anchored Proteins with Different GPI Attachment Signals form Distinct Clusters

Since it was proved that the expressed HRP-GPIs are applicable to the EMARS reaction, we employed this system to explore the co-clustering molecules with the HRP-DAFGPI and HRP-Thy1GPI. For identification of fluorescein-labeled molecules by the EMARS reaction, receptor tyrosine kinases (RTKs) antibody array was used as previously reported [23], [26], [27], [28]. The fluorescein-labeled RTKs in resting cells (*non-crosslinked*) were different between HRP-DAFGPI and HRP-Thy1GPI expressing cells (Figure 5C). These indicate that GPI-anchored proteins with different GPI-attachment signals form distinct clusters.

Lipid raft domains dynamically change after stimulation. They are on a small scale of less than 20 nm in resting cells, but they become on a large scale of more than 100 nm upon stimulation [2]. Therefore, we further investigated which molecules are co-clustered around the HRP-GPI molecules after crosslinking them with anti-HRP antibody. To this end, HRP-DAFGPI and HRP-Thy1GPI expressing HeLa S3 cells were separately reacted with anti-HRP antibody, and then subjected to the EMARS reaction and the RTKs antibody array. Expectedly the molecular species and intensity of fluorescein-labeled RTKs were increased after crosslinking (*crosslinked*) as compared to resting cells (*non-crosslinked*) in both types of HRP-GPI expressing cells (Figure 5C). In addition, the patterns of fluorescein-labeled RTKs after crosslinking (*crosslinked*) were also substantially different between HRP-DAFGPI and HRP-Thy1GPI expressing cells (Figure 5C). These results indicate that HRP-DAFGPI and HRP-Thy1GPI form different molecular clusters upon stimulation, too. Thus, molecular clusters containing distinct GPI-anchored proteins can be distinguished by using the EMARS method.

When cholesterol was depleted (*+MβCD; +Zaragozic acid and Simvastatin*), the signal intensities of fluorescein-labeled RTKs were considerably decreased in both types of HRP-GPI expressing cells (Figure 5C). This result supports that HRP-GPIs co-cluster with RTKs in lipid rafts.

In order to elucidate the effect of expression level of HRP-GPI on the clustering, HRP-DAFGPI was differentially expressed using different concentrations of the inducer, doxycycline (Figure 5D). As shown in Figure 5E, the expression level of HRP-DAFGPI affected the labeling intensity but hardly influenced the species of clustered molecules.

### Crucial Region in the GPI Attachment Signal that Affects N-glycosylation and Cluster Formation of GPI-anchored Proteins

**Figure 6:**
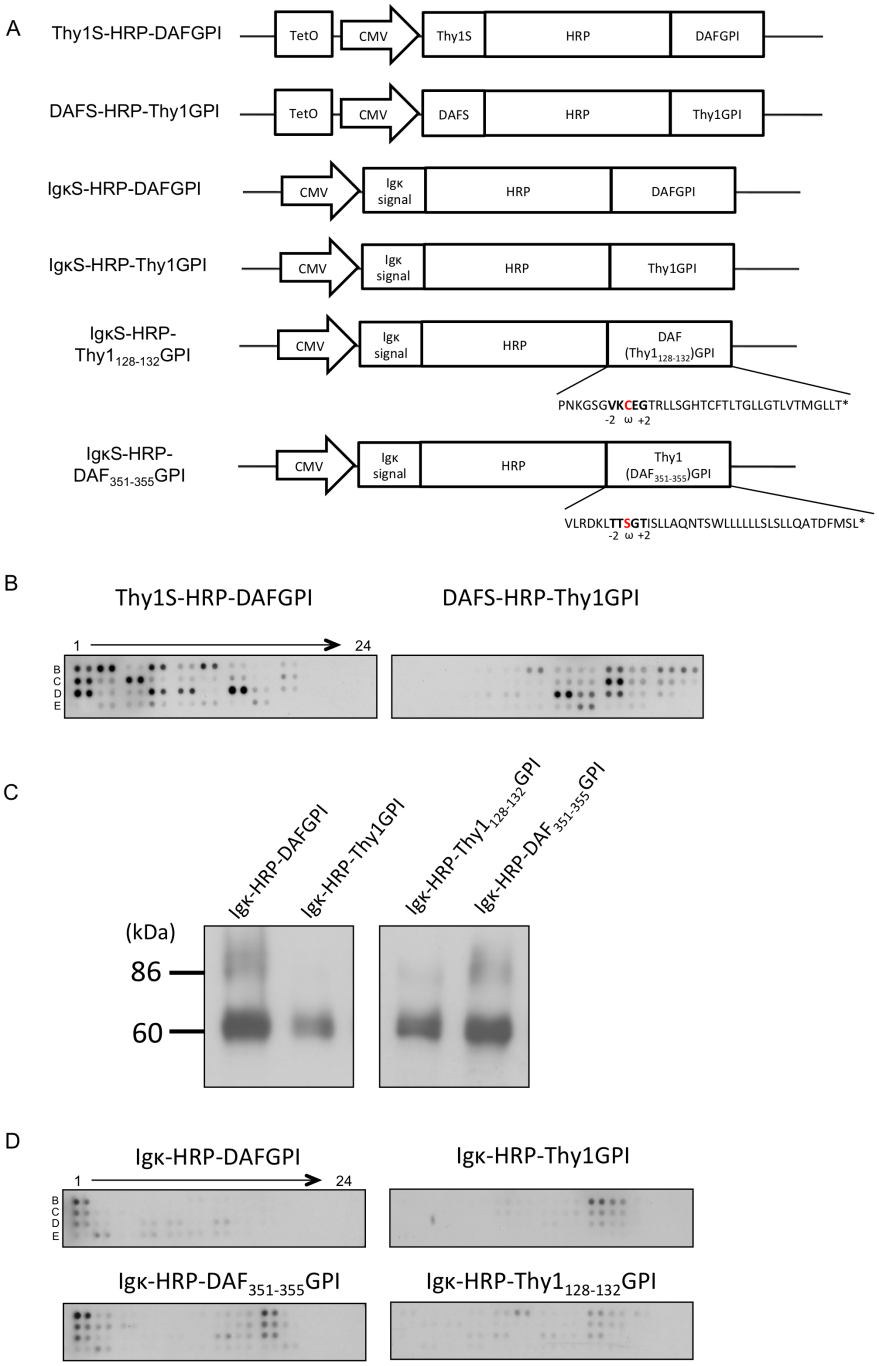
Effects of the GPI attachment signals on *N*-glycosylation and cluster formation of GPI-anchored proteins. *(A)* The swapped HRP-GPI constructs. Thy1S-HRP-DAFGPI encodes an HRP fusion protein with Thy1’s N-terminal signal sequence and DAF’s C-terminal GPI attachment signal. DAFS-HRP-Thy1GPI encodes an HRP fusion protein with DAF’s N-terminal signal sequence and Thy1’s C-terminal GPI attachment signal. Expression of Thy1S-HRP-DAFGPI and DAFS-HRP-Thy1GPI was driven by the doxycycline-dependent TetO promoter combined with the CMV promoter. IgκS-HRP-DAFGPI and IgκS-HRP-Thy1GPI encode HRP fusion proteins with the Ig κ chain’s N-terminal signal sequence *(Igκ signal)* and GPI attachment signals, *DAFGPI* and *Thy1GPI*. IgκS-HRP-Thy1_128–132_GPI and IgκS-HRP-DAF_351–355_GPI encode HRP fusion proteins with the Ig κ signal and partially swapped GPI attachment signals, *DAF(Thy1_128–132_)GPI* and *Thy1(DAF_351–355_)GPI*. DAF(Thy1_128–132_)GPI is designed as the five amino acids *(ω-2 to ω+2 site, bold)* of DAF’s GPI attachment signal are replaced with the corresponding five amino acids of Thy1’s. Thy1(DAF_351–355_)GPI is designed as the five amino acids *(ω-2 to ω+2 site, bold)* of Thy1’s are replaced with the corresponding five amino acids of DAF’s. The *ω sites* are shown in *red*. *(B)* Effect of the N-terminal signal sequences on cluster formation of GPI-anchored proteins. HeLa S3 cells that express Thy1S-HRP-DAFGPI or DAFS-HRP-Thy1GPI were reacted with the anti-HRP antibody to crosslink the HRP-GPIs, and then subjected to the EMARS reaction. The EMARS products (10 μg total protein) were applied to the RTKs antibody array. *(C)* IgκS-HRP-DAFGPI, IgκS-HRP-Thy1GPI, IgκS-HRP-Thy1_128–132_GPI or IgκS-HRP-DAF_351–355_GPI was transfected into HeLa S3 cells and transiently expressed. Cell lysates were subjected to SDS-PAGE and analyzed by Western blotting using an anti-HRP antibody. *(D)* Comparison of the EMARS products by antibody array analysis. HeLa S3 cells that transiently express IgκS-HRP-DAFGPI, IgκS-HRP-Thy1GPI, IgκS-HRP-DAF_351–355_GPI or IgκS-HRP-Thy1_128–132_GPI were treated with the anti-HRP antibody to crosslink the HRP-GPIs, and the EMARS reaction was performed. The EMARS products (40 μg total protein) were applied to the RTKs antibody array.

First, to exclude the possibility that the N-terminal signal sequences of HRP-GPIs influence their molecular cluster formation, we generated constructs in which the N-terminal signal sequences of HRP-DAFGPI and HRP-Thy1GPI were swapped each other, yielding Thy1S-HRP-DAFGPI and DAFS-HRP-Thy1GPI (Figure 6A). HeLa S3 cells expressing Thy1S-HRP-DAFGPI or DAFS-HRP-Thy1GPI were separately reacted with anti-HRP antibody, and then subjected to the EMARS reaction. The fluorescein-labeled EMARS products were identified using the RTKs antibody array. As the result, the patterns of fluorescein-labeled RTKs were quite similar between HRP-DAFGPI and Thy1S-HRP-DAFGPI, and also between HRP-Thy1GPI and DAFS-HRP-Thy1GPI expressing cells (compare Figure 5C, *crosslinked* and Figure 6B). This result indicates that the N-terminal signal sequences of HRP-GPIs are irrespective of their cluster formation, implying the contribution of the C-terminal GPI attachment signals.

Next, to determine the crucial region in the GPI attachment signals that causes differences in the *N*-glycosylation and the cluster formation of HRP-GPIs, the five amino acids (position ω-2 to ω+2) of the GPI attachment signals of Thy1_128–132_ (VKCEG) and DAF_351–355_ (TTSGT) were swapped each other in IgκS-HRP-DAFGPI and IgκS-HRP-Thy1GPI, yielding IgκS-HRP-Thy1_128–132_GPI and IgκS-HRP-DAF_351–355_GPI, respectively (Figure 6A). These four constructs have the same N-terminal signal sequence of the immunoglobulin κ chain and transiently expressed in HeLa S3 cells. When expressed HRP-GPIs were examined by Western blotting with an anti-HRP antibody, a band of 60 kDa was detected in the IgκS-HRP-Thy1_128–132_GPI transfectant corresponding to the IgκS-HRP-Thy1GPI one, whereas heterogeneous bands around 86 kDa were observed in the IgκS-HRP-DAF_351–355_GPI transfectant corresponding to the IgκS-HRP-DAFGPI one (Figure 6C).

We further investigated the molecular clusters of IgκS-HRP-Thy1_128–132_GPI and IgκS-HRP-DAF_351–355_GPI upon stimulation with anti-HRP antibody by using a combination of the EMARS reaction and RTKs antibody array analysis. As shown in Figure 6D, the pattern of fluorescein-labeled RTKs in the IgκS-HRP-DAF_351–355_GPI sample was similar to that of IgκS-HRP-DAFGPI rather than IgκS-HRP-Thy1GPI, while the pattern of IgκS-HRP-Thy1_128–132_GPI was like that of IgκS-HRP-Thy1GPI rather than IgκS-HRP-DAFGPI.

These results indicate that the five amino acids around the ω site are the crucial region in the GPI attachment signal to cause differences in the *N*-glycosylation and the cluster formation of HRP-GPIs. As the C-terminal side of the ω site is cleaved off upon the transfer of GPI-anchor, the three amino acids including the ω amino acid are assumed to be a minimum requirement for the effects.

## Discussion

The original version of the EMARS method employs HRP-conjugated antibodies for the activation of an arylazide compound [23]. Since many HRP-conjugated antibodies are commercially available, this system is simple and convenient but has some limitations: first, a proper HRP-conjugated antibody for the probed molecule is not always available; second, only cell surface clusters can be examined because HRP-conjugated antibodies cannot enter the cell; third, crosslinking with antibodies may cause artificial cluster formation. In the present study, we have established a new version of the EMARS method, in which the EMARS reaction is catalyzed by intracellularly expressed HRP fusion proteins in substitution for exogenously added HRP-conjugated antibodies. HRP can be fused to any protein of interest. We have applied this strategy to identify co-clustered molecules with GPI-anchored proteins that are a well-characterized component of lipid rafts.

Expression of HRP in mammalian cells was first introduced in a study with regard to intracellular protein transport [35]. Thereafter, use of HRP and its chimeric proteins as a reporter gene has increased in studies on neural tract tracing and ultrastructural observation with electron microscopy [36], [37]. HRP was transiently expressed in these studies, and serious toxicity due to expressed HRP was not seen. In the present study, HRP was expressed in a GPI-anchored form for the first time. During the screening of stable transfectants, we noticed that sustained expression of HRP caused morphological changes and cell death although the reason is unknown. Therefore, an inducible expression system using the Tet-On system was needed to obtain stable transfectants. Eventually, GPI-anchored HRP was successfully expressed in human cells, retaining its activity by the induction of its transcription.

**Figure 7:**
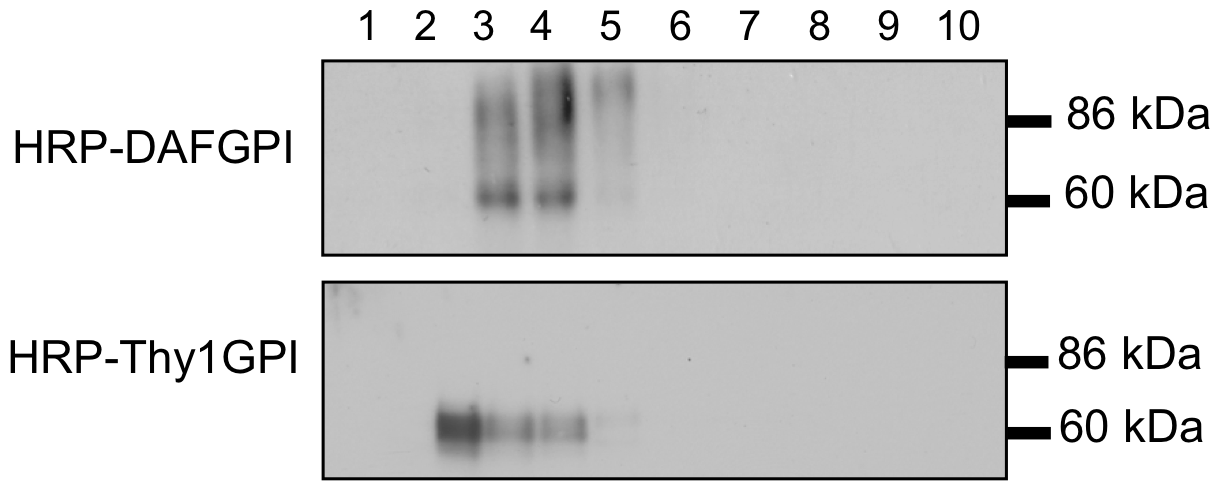
Sedimentation velocity of HRP-GPIs in a sucrose density gradient ultracentrifugation. HeLa S3 cells that express HRP-DAFGPI or HRP-Thy1GPI were lysed in buffer containing 0.4% SDS and 0.2% TtitonX-100 and run through 5–30% sucrose gradients. Fractions of 1 ml were collected from the top (fraction1) to the bottom (fraction10) of the gradients. HRP-GPIs were detected by Western blotting using an anti-HRP antibody.

The native *Armoracia rusticana* HRP has a molecular mass of 42–44 kDa and contains eight *N*-linked glycans accounting for 22–27% of its molecular mass [38]. When HRP was expressed in a GPI-anchored form in human HeLa S3 cells in this study, its molecular mass was much larger than 44 kDa (Figure 3A), implying differences in the structure of *N*-glycan chains between plant and mammal. Plant *N*-glycans contain α1-3-linked fucose and xylose that are not found in mammalian *N*-glycans, but lack galactose and sialic acid residues that are usually seen in mammalian *N*-glycans. Furthermore, there was a remarkable difference in the *N*-glycan structures between HRP-DAFGPI and HRP-Thy1GPI. HRP-DAFGPI had complex type oligosaccharides containing sialic acid, while HRP-Thy1GPI had high mannose type oligosaccharides (Figure 3B). Reflecting this difference, the molecular mass of HRP-DAFGPI was larger than that of HRP-Thy1GPI on SDS-PAGE (Figure 3A). This result was supported by the velocity gradients analysis, in which HRP-DAFGPI was recovered in slightly heavier fractions than HRP-Thy1GPI (Figure 7). This difference in *N*-glycan structures did not affect the enzyme activity of the expressed HRPs so much (Figure 5A).

**Figure 8:**
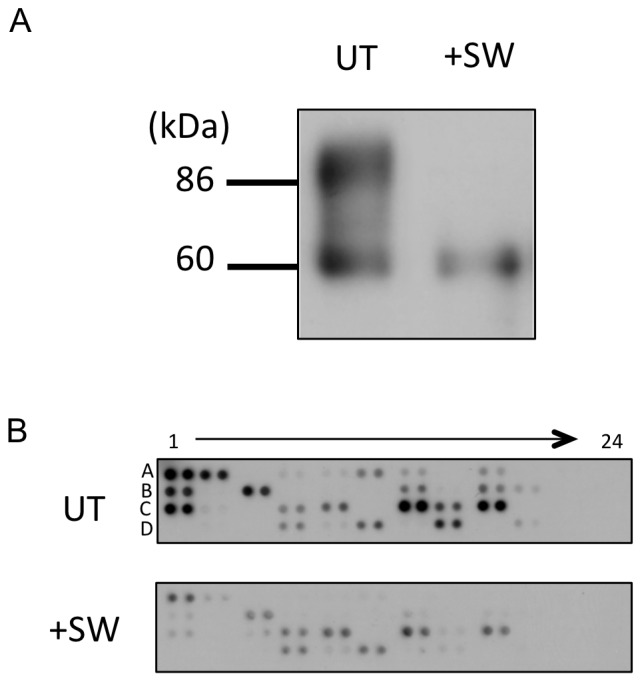
Effects of *N*-glycan processing on cluster formation of GPI-anchored proteins. (*A*) HRP-DAFGPI-expressing cells were treated with (+*SW)* or without (*UT)* 20 μM swainsonine. Cell lysates were subjected to Western blotting using anti-HRP antibody. (*B*) Identification of the fluorescein-labeled EMARS products by the RTKs antibody array analysis. After treatment with (+*SW)* or without (*UT)* swainsonine, HeLa S3 cells that express HRP-DAFGPI were crosslinked with an anti-HRP antibody and subjected to the EMARS reaction. Cell membrane extracts were applied to an RTKs antibody array and EMARS reaction products were detected with an anti-fluorescein antibody.

Considering that the processing of *N*-glycan chains occurs in the Golgi apparatus, it is suggested that HRP-DAFGPI and HRP-Thy1GPI are differently sorted before they are transported into the Golgi apparatus. When the HRP-DAFGPI-expressing cells were treated with swainsonine, which prevents synthesis of the complex type *N*-glycans by inhibiting Golgi α-mannosidase II, the 86 kDa band of HRP-DAFGPI disappeared (Figure 8A), indicating that the swainsonine-treated HRP-DAFGPI carries only high-mannose type *N*-glycans like HRP-Thy1GPI (Figure 3). Nevertheless, the pattern of co-clustered molecules with the swainsonine-treated HRP-DAFGPI was similar to that with the untreated HRP-DAFGPIs rather than that with HRP-Thy1GPI (Figure 8B). This result suggests that the microenvironment for cluster formation of HRP-GPIs is generated independently of *N*-glycosylation. These findings, therefore, support the hypothesis that lipid raft formation initiates in the early stage of intracellular trafficking, in good agreement with the finding that cholesterol depletion affects oligomerization of GPI-anchored proteins in the Golgi apparatus but does not dissociate once formed oligomers [39], [40]. Oligomerization of GPI-anchored proteins, which is a prerequisite for the apical sorting, depends on cholesterol content in Madin-Darby canine kidney cells [22] and *N*-glycosylation of GPI-anchored proteins in Fisher rat thyroid cells [40]. Paladino et al. demonstrated that different GPI-attachment signals affect the oligomerization of GPI-anchored proteins and their intracellular trafficking [22]. In this study, however, neither HRP-DAFGPI nor HRP-Thy1GPI formed oligomer (Figure 7), indicating that oligomerization is not required for the cluster formation of these HRP-GPIs in non-polarized HeLa S3 cells. Considering that oligomerization is needed for apical sorting in polarized epithelial cells [22], [39], [40], the clustering mechanism could be different between polarized and non-polarized cells. Since basolateral GPI-anchored proteins are also raft associated [39], [40], lipid raft formation might be underlied by a different mechanism from oligomerization.

The molecular species and amount of labeled RTKs increased by crosslinking of HRP-GPIs with anti-HRP antibody (Figure 5C), indicating that molecular clustering was enhanced by the crosslinking. Since GPI is associated with the lipid rafts, crosslinking of GPI-anchored proteins may perturb the membrane structures extensively. In fact, the crosslinking of GPI-anchored proteins, such as CD59, DAF, and Thy-1, induces the formation of molecular complexes with Src family kinases and G protein-coupled receptors and their activation [41], [42], [43], [44], [45]. Alternatively, the reduction of diffusion rate of GPI-anchored protein by crosslinking [45] may cause the signal intensification. The expression level of HRP-GPI enhanced the labeling intensity but did not affect the molecular species of clustered molecules (Figure 5E).

The effect of crosslinking with antibodies is also observed in the EMARS system. A wide range of RTKs were intensely labeled when an intact anti-β1 integrin antibody and an HRP-conjugated second antibody are used for the probe of the EMARS reaction [23]. By contrast, only specific RTKs were labeled when an HRP-conjugated monovalent anti-β1 integrin antibody was used [27]. From this viewpoint, the use of expressed HRP in the EMARS reaction is better than HRP-conjugated antibodies in that expressed HRP can evade the artificial cluster formation by antibodies, reflecting a natural state in living cells.

GPI-anchored proteins are considered to interact with each other and with other molecules in lipid rafts via the GPI anchor and/or the protein ectodomain, in which lipid-lipid, lipid-protein and protein-protein interactions are involved [22]. In the swapping experiment of the GPI attachment signals (Figure 6), the three amino acids (ω-2 to ω) in the linker region were found to be responsible for differences in glycosylation and cluster formation of HRP-GPIs. Important questions remain to be solved. Which of the proximal linker region or the GPI moiety is directly involved in specific cluster formation of GPI-anchored proteins? Does the proximal linker region dictate the remodeling of GPI anchors in the Golgi apparatus? Is the GPI remodeling associated with the processing of *N*-glycan? Further studies are needed to characterize the structural composition of GPI anchors of distinct GPI-anchored proteins.

In conclusion, the new version of EMARS method using expressed HRP fusion proteins can identify co-clustering molecules in individual lipid raft domains under a physiological condition. This new approach will provide a useful tool for a wide range of research concerning molecular interactions within the cells as well as on the cell surface.
